# Immunization with the receptor-binding domain of SARS-CoV-2 elicits antibodies cross-neutralizing SARS-CoV-2 and SARS-CoV without antibody-dependent enhancement

**DOI:** 10.1038/s41421-020-00199-1

**Published:** 2020-09-03

**Authors:** Jinkai Zang, Chenjian Gu, Bingjie Zhou, Chao Zhang, Yong Yang, Shiqi Xu, Lulu Bai, Rong Zhang, Qiang Deng, Zhenghong Yuan, Hong Tang, Di Qu, Dimitri Lavillette, Youhua Xie, Zhong Huang

**Affiliations:** 1 CAS Key Laboratory of Molecular Virology & Immunology, Institut Pasteur of Shanghai, Center for Biosafety Mega-Science, Chinese Academy of Sciences, University of Chinese Academy of Sciences, Shanghai 200031, China; 2 Key Laboratory of Medical Molecular Virology (MOE/NHC/CAMS), Department of Medical Microbiology and Parasitology, School of Basic Medical Sciences, Shanghai Medical College, Fudan University, Shanghai 200031, China; 3 BSL-3 Laboratory of Fudan University, School of Basic Medical Sciences, Shanghai Medical College, Fudan University, Shanghai 200032, China

**Keywords:** Immunology, Biological techniques

Dear Editor,

The ongoing coronavirus disease 2019 (COVID-19) pandemic is a serious public health crisis, which is caused by severe acute respiratory syndrome coronavirus 2 (SARS-CoV-2). The major structural protein of SARS-CoV-2 is spike (S) protein, and its ectodomain is divided into two subunits, S1 and S2, which are responsible for receptor binding and membrane fusion, respectively. Like the closely related severe acute respiratory syndrome coronavirus (SARS-CoV), SARS-CoV-2 uses human angiotensin-converting enzyme 2 (ACE2) as entry receptor^1^. S protein binds ACE2 through its receptor-binding domain (RBD) located within S1 subunit.

Thus far, a number of SARS-CoV-2 vaccine candidates derived from different vaccine platforms, including DNA vaccine, mRNA vaccine, inactivated whole virus vaccine, and adenovirus-vectored vaccine, have rapidly progressed into clinical trials^2,3^. One of the challenges in developing vaccines for coronaviruses is the potential vaccine-induced immune enhancement of disease^4,5^. Antibodies raised against inactivated whole-virion coronavirus vaccine, especially antibodies targeting S protein, may increase viral infection of Fc receptor (FcR)-expressing cells—a phenomenon called antibody-dependent enhancement (ADE), which is well documented for flaviviruses^4,5^. So far, all SARS-CoV-2 vaccine candidates entering clinical trials contain or express full-length or near full-length S protein and therefore bear risk of ADE. Thus, it is important to continue the search for a safe and effective SARS-CoV-2 vaccine.

Recombinant RBD proteins of SARS-CoV and MERS-CoV have been shown to potently induce protective neutralizing antibodies and are therefore considered promising vaccine candidates^6,7^. In this study, we evaluated the possibility of developing SARS-CoV-2 RBD (hereafter referred as SARS2-RBD)-based vaccines. Specifically, we investigated whether recombinant SARS2-RBD could elicit in mice neutralizing antibodies and whether such antibodies could promote ADE in vitro.

To rapidly evaluate vaccine potential of SARS2-RBD, a pilot mouse immunization study was performed with recombinant RBD/mouse IgG1-Fc fusion protein (RBD-Fc) as immunogen. The mice received three doses at days 0, 8, and 13. One week after the last immunization, antisera were collected from the three immunized mice for antibody measurement. All three antisera dose-dependently reacted with His-tagged SARS2-RBD in ELISA, whereas control sera from a naïve mouse did not show significant reactivity (Supplementary Fig. S1a). Anti-RBD-Fc sera #1 with the highest RBD-binding titer (2 × 10^5^) was selected for further analyses. Anti-RBD-Fc sera #1 dose-dependently inhibited binding between recombinant ACE2-Fc fusion protein and His-tagged SARS2-RBD in competition ELISA (Supplementary Fig. S1b), indicating that the antisera contain antibodies targeting receptor-binding motif (RBM) within RBD. Anti-RBD-Fc sera #1 was then assessed for the ability to neutralize SARS-CoV-2 pseudovirus (hereafter referred as SARS2-PV). The antisera dose-dependently neutralized SARS2-PV entry with a calculated 50% neutralization titer (NT50) value of 10,513 (Supplementary Fig. S1c). Moreover, anti-RBD-Fc sera #1 were highly effective on neutralizing authentic SARS-CoV-2, based on qRT-PCR and immunofluorescent analyses (Supplementary Fig. S1d and e). These results demonstrate that RBD-Fc is an immunogen capable of efficiently inducing SARS-CoV-2-neutralizing antibodies.

To verify that the RBD part within RBD-Fc fusion protein is indeed responsible for induction of neutralizing antibodies against SARS-CoV-2, we performed a second mouse immunization study with recombinant SARS-CoV-2 RBD as vaccine antigen. A group of BALB/c mice were immunized with recombinant RBD at days 1, 10, and 25 (Fig. 1a). Another group of mice were injected with an irrelevant protein (HBc, hepatitis B virus core protein), serving as control. Antisera were collected from individual mice at days 20 and 40, and analyzed for SARS2-RBD-specific antibody by ELISA. As shown in Fig. 1b, neither the day-20 nor the day-40 sera in control group exhibited any significant binding activity; in contrast, SARS2-RBD-binding activity was readily detectable for the day-20 anti-RBD sera and a significant increase in SARS2-RBD-binding was observed for the day-40 anti-RBD sera. Equal amount of individual antisera in the same groups were pooled for subsequent analyses. The day-20 and day-40 pooled anti-RBD sera dose-dependently reacted with SARS2-RBD in ELISAs (Fig. 1c) and their binding antibody titers were determined to be 1.6 × 10^5^ and 3.2 × 10^6^, respectively. SARS2-RBD-binding activity of anti-RBD sera collected at day 60 (when the mice were euthanized) was comparable to that of the day-40 anti-RBD sera (Supplementary Fig. S2).

**Fig. 1:**
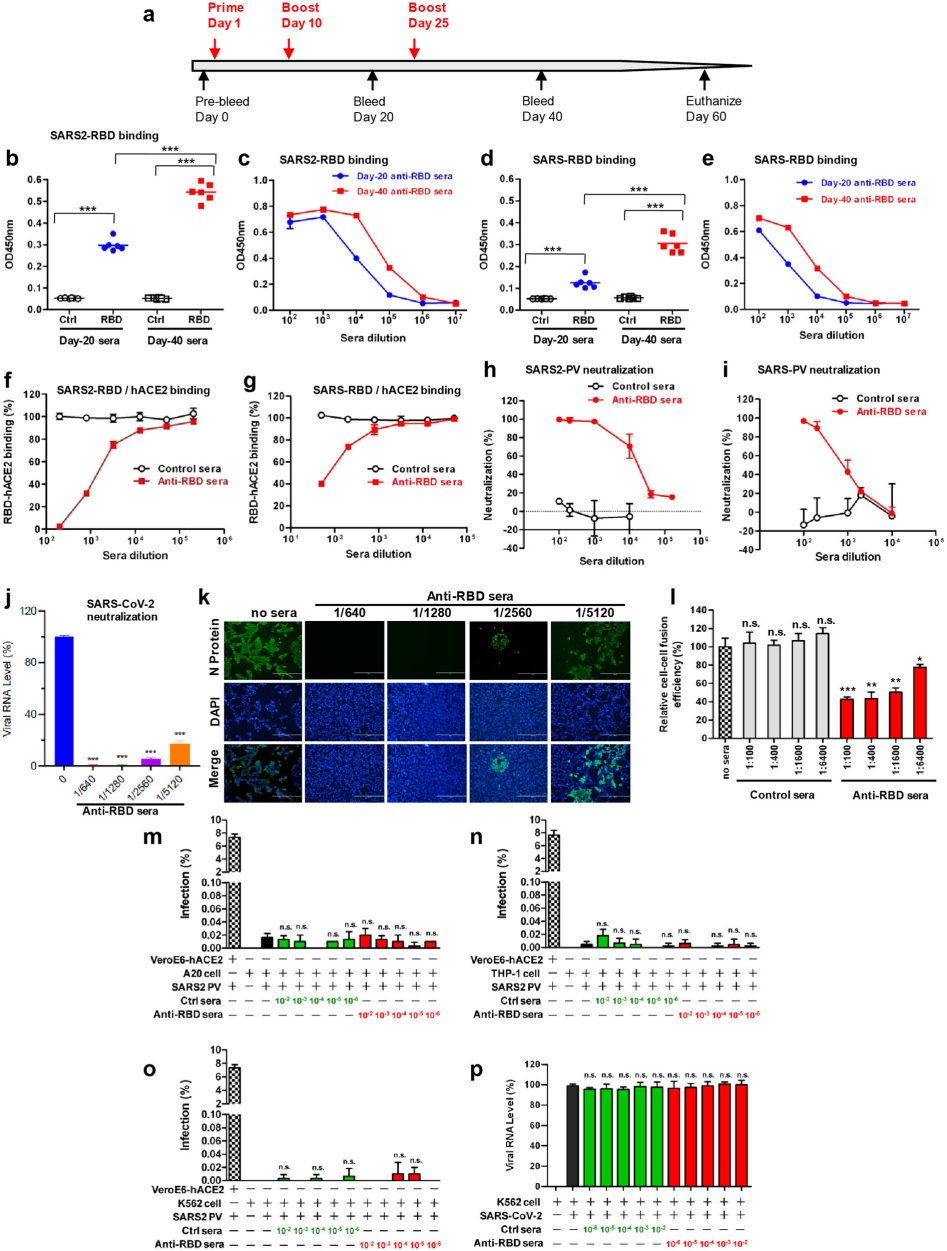
Antibodies elicited by recombinant SARS2-RBD cross-neutralized SARS-CoV-2 and SARS-CoV without ADE. **a** Mouse immunization and sampling schedule. **b**, **c** SARS2-RBD-binding activities of individual (**b**) and pooled (**c**) anti-RBD antisera. **d**, **e** SARS-RBD-binding activities of individual (**d**) and pooled (**e**) anti-RBD antisera. For **b** and **d**, sera were diluted 1:10,000, and each symbol represents a mouse and the line indicates geometric mean value. Significant differences between groups were indicated: ***P < 0.001. **f**, **g** Blockade of ACE2 binding to immobilized SARS2-RBD (**f**) and SARS-RBD (**g**) by pooled anti-RBD sera. **h**, **i** Anti-RBD sera (day-40 pooled sera) neutralized SARS2-PV (**h**) and SARS-PV (**i**) in vitro. **j** Neutralization efficiency of anti-RBD sera against authentic SARS-CoV-2. Viral RNA copy number was determined by qRT-PCR. **k** Neutralization of authentic SARS-CoV-2 revealed by immunofluorescent staining. Scale bars, 400 μm. **l** Inhibitory effect of serially diluted anti-RBD sera or control sera on SARS2-S-mediated cell–cell fusion. For a given sample, its cell–cell fusion efficiency (ratio of dual-fluorescence cells to EGFP-only cells) was normalized against that of the sample without antisera treatment. **m**–**o** ADE assays with SARS2-PV. The sera/SARS2-PV mixtures were added to A20 (**m**), THP-1 (**n**), or K562 (**o**) cell suspensions. After incubation, infected cells were analyzed by flow cytometry. Data are expressed as percentage of GFP-expressing cells in relation to total cells counted. **p** ADE assay with live SARS-CoV-2. The sera/live virus mixtures were added to K562 cell suspensions. After incubation, infected cell cultures were subjected to qRT-PCR analysis. For **j** and **p**, data are expressed as percentage of viral RNA copy number of the treatment groups in relation to that of the virus-only control. For **c**, **e**–**g**, **j**, **l**–**p**, means ± SD of triplicate wells are shown. For **h** and **i**, data (means ± SD) from three independent experiments are shown. For **j**, **l**–**p**, significant differences between the virus-only group and each treatment group were indicated: n.s., *P* > 0.05; *P < 0.05; **P < 0.01; ***P < 0.001.

SARS2-RBD shares high homology with SARS-CoV RBD (hereafter referred as SARS-RBD) in sequence. This prompted us to evaluate the cross-reactivity of SARS2-RBD-immunized sera towards SARS-RBD. As shown in Fig. 1d, e, both individual and pooled sera from SARS2-RBD-immunized mice showed dose-dependent binding activity with SARS-RBD. SARS-RBD-binding titers of the pooled day-20 and day-40 anti-RBD sera were determined to be 4 × 10^3^ and 1.6 × 10^5^, respectively.

The pooled day-40 antisera were assessed for their ability to block interaction between RBDs and human ACE2. ELISA results showed that the day-40 anti-RBD sera, but not control sera, dose-dependently inhibited hACE2-Fc binding to SARS2-RBD (Fig. 1f). Anti-RBD sera also exhibited blockade effect on the SARS-RBD/hACE2-Fc interaction, albeit with a lower efficiency (Fig. 1g).

Neutralization capacity of mouse antisera was first evaluated using SARS2-PV. The day-40 anti-RBD sera potently inhibited SARS2-PV infection and the calculated NT50 was 12,764 (Fig. 1h). The same anti-RBD sera also inhibited infection of SARS-CoV pseudovirus (SARS-PV) with NT50 being 834.8 (Fig. 1i). Anti-RBD sera, but not control sera, were also found to potently inhibit authentic SARS2-CoV-2 infection based on cytopathic effect (CPE) observation (Supplementary Fig. S3). qRT-PCR and IFA assays revealed that anti-RBD sera diluted 1:1280 almost completely blocked viral infection and even 1:5120 diluted anti-RBD sera inhibited viral infection by 83% (Fig. 1j, k). These results demonstrate that anti-RBD sera possessed strong neutralization capacity against SARS-CoV-2.

SARS-CoV-2 S protein has been shown to bind cell-surface ACE2 and mediate cell–cell fusion, leading to syncytia formation^8^. A cell–cell fusion assay was developed to determine whether anti-RBD sera could prevent S-mediated syncytia formation. Co-culture of 293 T cells expressing S:EGFP fusion protein and 293 T cells expressing human ACE2 fused with mCherry (hACE2:mCherry) led to detection of dual-fluorescent cells, indicating occurrence of cell–cell fusion (Supplementary Fig. S4). The cells solely emitting green or red fluorescence and the dual-fluorescent cells were quantified by flow cytometry. Treatment with the day-40 anti-RBD antisera but not control sera significantly inhibited cell–cell fusion in an antisera dose-dependent manner (Fig. 1l and Supplementary Fig. S5). Thus, anti-RBD sera are able to inhibit SARS2-S-mediated cell–cell fusion. Moreover, the day-40 anti-RBD antisera were also able to inhibit SARS-S-mediated cell–cell fusion, albeit with a lower efficiency (Supplementary Fig. S6).

We then evaluated the ADE potential of the mouse antisera. Several FcR-bearing cell lines were used as target cells in ADE assays, including mouse A20 cells expressing FcγRII, human THP-1 cells expressing both FcγRI and FcγRII, and K562 cells expressing human FcγRII. THP-1 and K562 cells have been shown to support mouse antibody-mediated enhancement of dengue virus infection in previous studies^9,10^. We found that SARS2-PV entry into the three FcR-expressing cell lines was minimal (< 0.02%) whereas the same amount of SARS2-PV yielded an infection rate of ~7% in VeroE6-hACE2 cells. Moreover, treatment with serially diluted control sera or anti-RBD sera did not significantly affect SARS2-PV entry of the three cell lines (Fig. 1m–o), indicating that anti-RBD sera do not promote ADE of SARS2-PV. We selected K562 cells for ADE assay with authentic SARS-CoV-2. No significant increase in viral RNA level was observed for antisera-treated samples as compared to the virus-only control regardless of the antisera dilutions (Fig. 1p). These results demonstrate that anti-RBD antibodies do not promote ADE.

Our study demonstrated that anti-RBD sera exhibited potent neutralization effects on SARS-CoV-2. Moreover, anti-RBD sera inhibited SARS2-S-mediated cell–cell fusion. Importantly, anti-RBD antibodies do not promote ADE, at least not in the assay system we used. It remains to be determined whether antibodies targeting other regions of S protein could mediate ADE of SARS-CoV-2. Interestingly, anti-SARS2-RBD sera were found to cross-react with SARS-RBD. Binding titers to SARS-RBD were 20–40 fold lower than that to SARS2-RBD, probably due to the variation in RBD sequence between SARS-CoV and SARS-CoV-2. RBM varies significantly (~47% homology in amino acid sequence) between SARS-CoV and SARS-CoV-2 despite the core subdomain is highly conserved. Therefore, the observed cross-binding towards SARS-RBD and cross-neutralization of SARS-CoV is likely contributed by antibodies targeting the conserved SARS-CoV-2 core subdomain, which contains cross-neutralization antibody epitopes. Our work provides important information for further development of RBD-based SARS-CoV-2 or pan-SARS-CoV subunit vaccines.

## Supplementary information

Supplementary Information

## Supplementary information

**Supplementary Information** accompanies the paper at (10.1038/s41421-020-00199-1).
