# Supplementary material for: Immunization with the receptor-binding domain of SARS-CoV-2 elicits antibodies cross-neutralizing SARS-CoV-2 and SARS-CoV without antibody-dependent enhancement
Source: Cell Discov. 2020 Sep 3;6:61. doi: 10.1038/s41421-020-00199-1 (PMC7471522; doi:10.1038/s41421-020-00199-1)
Supplement: Supplementary file 1 — Supplementary Information [file 41421_2020_199_MOESM1_ESM.pdf]

Supplementary Information

Immunization with the receptor–binding domain of SARS-CoV-2 elicits antibodies cross-neutralizing SARS-CoV-2 and SARS-CoV without antibody-dependent enhancement

Jinkai Zang[1, #], Chenjian Gu[2, #], Bingjie Zhou[1, #], Chao Zhang[1, #], Yong Yang[1, #], Shiqi Xu[1], Lulu Bai[1], Rong Zhang[2], Qiang Deng[2], Zhenghong Yuan[2], Hong Tang[1], Di Qu[3], Dimitri Lavillette[1,*], Youhua Xie [2, *], Zhong Huang [1,*]

[1] CAS Key Laboratory of Molecular Virology & Immunology, Institut Pasteur of Shanghai, Center for Biosafety Mega-Science, Chinese Academy of Sciences, University of Chinese Academy of Sciences, Shanghai 200031, China; [2] Key Laboratory of Medical Molecular Virology (MOE/NHC/CAMS), Department of Medical Microbiology and Parasitology, School of Basic Medical Sciences, Shanghai Medical College, Fudan University, Shanghai 200032, China; [3] BSL-3 Laboratory of Fudan University, School of Basic Medical Sciences, Shanghai Medical College, Fudan University, Shanghai 200032, China

[#] These authors contributed equally: Jinkai Zang[1, #], Chenjian Gu[2, #], Bingjie Zhou[1, #], Chao Zhang[1, #], Yong Yang[1, #]

* Corresponding author:

Prof., Dr. Zhong Huang (huangzhong@ips.ac.cn)

Prof., Dr. Youhua Xie (yhxie@fudan.edu.cn)

Prof., Dr. Dimitri Lavillette (dlaville@ips.ac.cn)

Materials and Methods

Cells and viruses

VeroE6 cells were grown as described previously [1]. HEK 293T, A20 [2], THP-1 [3], and K562 cells [4] were purchased from the Cell Bank of Chinese Academy of Sciences (www.cellbank.org.cn). A clinical isolate of SARS-CoV-2, nCoV-SH01 (GenBank: MT121215.1) [5], was propagated in VeroE6 cells and viral titer was determined as plaque forming units (PFU) per milliliter (mL) by CPE quantification. Live virus infection experiments were performed in the biosafety level-3 (BSL-3) laboratory of Fudan University.

Recombinant proteins

For mouse immunization, recombinant SARS-CoV-2 RBD fusion protein with the Fc region of mouse IgG1 at the C-terminus (RBD-Fc) was purchased from Sino Biological (Beijing, China), recombinant SARS-CoV-2 RBD with a C-terminal His-tag was purchased from Kactus Biosystems (Shanghai, China), and recombinant hepatitis B core antigen (HBc) was produced in house in *E.coli* as described previously [6]. For biochemical and immunological assays, several mammalian cell-produced recombinant proteins were generated in house, including SARS-CoV-2 RBD (amino acids 320 to 550) fused with an N-terminal Strep-tag and a C-terminal His-tag, SARS-CoV RBD (amino acids 306-520) fused with a C-terminal His-tag, and human ACE2 ecotodomain fused with human IgG1 Fc at the C-terminus (hACE2-Fc). Biotinylated hACE2-Fc was prepared using EZ-Link™ Sulfo-NHS-LC-LC-Biotin kit (Thermo Fisher Scientific).

Mouse immunization

All the animal experiments in this study were approved by the Institutional Animal Care and Use Committee at the Institut Pasteur of Shanghai. Animals were cared for in accordance with institutional guidelines.

In the first immunization experiment, three BALB/c mice were each injected intraperitoneally (i.p.) with 100 μg of RBD-Fc fusion protein formulated with 500 μg of

aluminum hydroxide (Alhydrogel, Invivogen, USA) and 25 μg of CpG (Sangon, China) at day 0. The mice were boosted subcutaneously (s.c.) at day 8 with 50 μg of RBD-Fc plus Freund's Adjuvant Complete (Sigma, USA) and at day 13 with 50 μg of RBD-Fc plus Titermax adjuvant (Sigma). Blood were collected from individual mice one week after the last immunization (day 20) and sera were stored at -80 °C until use.

In the second immunization experiment, recombinant RBD protein containing a C-terminal 6xHis tag was formulated with the Alhydrogel adjuvant (Invivogen) and each vaccine dose contained 5 μg of RBD and 500 μg of aluminum hydroxide; in addition, a negative antigen control was prepared by mixing 5 μg of recombinant HBc protein with 500 μg of aluminum hydroxide. Two groups of six BALB/c mice were injected intraperitoneally (i.p.) with the RBD vaccine and the control antigen, respectively, at days 0, 10, and 25. Blood samples were collected from individual mice at days 20, 40 and 60 for antibody measurement.

Serum antibody measurement

For antibody measurement, wells of 96-well microtiter plates were coated with the indicated amounts of the SARS2-RBD or SARS-RBD recombinant protein for 2 hrs at 37 °C or overnight at 4°C. Then the wells were blocked with PBST containing 5% non-fat dry milk for 1 hr at 37℃, incubated with 50μl serially diluted mouse antisera for 2 hrs at 37°C and then with 50 μl of horseradish peroxidase (HRP)-conjugated goat anti-mouse IgG antibody for 1 hr 37°C. After color development, the absorbance at 450 nm was measured in a 96-well plate reader. For a given serum sample, its endpoint titer was reported as the reciprocal of the highest serum dilution that had an absorbance ≥0.1 OD unit above the blank.

ACE2 competition ELISA

Wells of 96-well microtiter plates were coated with 25 ng/well of the SARS2-RBD or SARS-RBD recombinant protein overnight at 4°C, followed by blocking with PBST containing 5% non-fat dry milk for 1 hr at 37℃. Serially diluted mouse antisera were mixed with 20 ng of biotinylated hACE2-Fc in a final volume of 50μl and the mixtures

were added to the wells, followed by incubation for 2 hrs at 37°C. Then, 50 μl of horseradish peroxidase (HRP)-conjugated streptavidin (Life Technologies, USA) was added to wells, followed by incubation for 1 hr at 37°C. After washing, TMB substrate (Life Technologies) was added into wells for color development. The plates were read for absorbance at 450 nm in a 96-well plate reader.

Cell-cell fusion inhibition assay

HEK 293T cells were separately transfected with a plasmid encoding the SARS-CoV-2 S:EGFP fusion protein (pcDNA-S:EGFP) or with a plasmid encoding the hACE2:mCherry fusion protein (pcDNA-hACE2:mCherry). One day later, equal amount of the pcDNA-S:EGFP-transfected and pcDNA-hACE2:mCherry-transfected cells were mixed and then cultured for 24 hrs. Unmixed cells were set aside as controls. To determine the antisera's blockade effects, pcDNA-S:EGFP-transfected cells were treated with serially diluted antisera for 1 hr at 37 °C before mixing with pcDNA-hACE2:mCherry-transfected cells. After co-culture for 24 hrs, the cells were subjected to fluorescence microscopy or flow cytometry. The cells emitting green or red fluorescence only or both were quantified by flow cytometry. For a given sample, its cell-cell fusion efficiency was calculated and normalized against that of the sample without antisera treatment as follows: relative cell-cell fusion efficiency (%) = (the ratio of the dual-fluorescence cells to the EGFP-only cells of the given sample) / (the ratio of the dual-fluorescence cells to the EGFP-only cells of the sample without antisera treatment)×100. For the SARS-CoV S-mediated cell-cell fusion assay, the same procedure as above was followed, except that a plasmid encoding the SARS-CoV S protein fused to EGFP (pcDNA-SARS-S:EGFP) was used.

Pseudovirus neutralization assay

To produce pseudoviruses, HEK293T cells were transfected using PEI with a plasmid encoding murine leukemia virus (MLV) gag/pol, a retroviral vector encoding EGFP, and an envelope plasmid expressing full-length S protein of SARS-CoV-2 or SARS-CoV (AY569693). Six hours later, the cells were washed and incubated in fresh

medium. At 48 hours post-transfection, pseudovirus-containing culture supernatants were harvested. For neutralization assay, 100 μl of the pseudovirus was pre-mixed with 50 μl of serum samples diluted in DMEM and incubated at 37 °C for 1 hr. The mixture was then onto VeroE6 cells overexpressing hACE2 (denoted as VeroE6-hACE2) preseeded in 48-well plates. Eight hours later, the virus/sera-containing media were removed and exchanged with fresh media containing 10% FBS. At 72 hours post-infection, the cells were analyzed by flow cytometry. The infectivity of pseudotyped particles incubated with antibodies was compared with the infectivity observed using pseudotyped particles incubated with DMEM medium containing 2% FBS and standardized to 100%.

Live virus neutralization assay

All serum samples were heat-inactivated at 56°C for 30 minutes prior to live virus neutralization assay. SARS-CoV-2 virus (200 PFU in a volume of 50μl) was pre-incubated with the diluted serum sample for 1 hour at 37°C. The virus-serum mixture was then added onto VeroE6 cells (4×10^4/well) in 96-well plate and cultured for 48 hours. At the end of the incubation, culture supernatants were collected for viral RNA analysis and cells were fixed for immunofluorescence analysis.

Viral RNA in culture supernatant was extracted using TRIzol reagent (Invitrogen, USA) following the manufacturer's instructions. Quantitative real-time PCR (qRT-PCR) was performed in a 20-μL reaction containing SYBR Green (Tiangen, China) on an MXP3000 cycler (Stratagene, La Jolla, USA). PCR primers (Genewiz, Suzhou, China) targeting SARS-CoV-2 N gene (nt 608-706) were as the following: forward primer, 5'-GGGGAACTTCTCCTGCTAGAAT-3'; and reverse primer, 5'-CAGACATTTTGCTCTCAAGCTG-3'.

For immunofluorescence analysis, cells were fixed in 4% paraformaldehyde, permeabilized by 0.2% Triton X-100 (Thermo Fisher Scientific, USA), and stained overnight at 4°C with an anti-N mouse polyclonal antibody generated in house. The samples were finally incubated with Alexa Fluor 488-labeled donkey anti-mouse IgG secondary antibody (1:1000, Thermo Fisher Scientific) at 37°C for 1 hour. The nuclei

were stained with DAPI (Thermo Fisher Scientific). Images were captured under a fluorescence microscope (Thermo Fisher Scientific).

ADE assay

FcR-expressing cell lines, including A20, THP-1, and K562, were used to perform ADE assays. Briefly, the antisera were serially diluted, mixed with either SARS2-PV or authentic SARS-CoV-2 (6,000 PFU), and incubated at 37℃ for 1 hr. Then, the mixtures were added to the target cells. The following infection and culturing steps were carried out as described above in the pseudovirus neutralization and live virus neutralization assays. Mock-infected cells and cells only infected with SARS2-PV or authentic SARS-CoV-2 were set as the negative and positive controls, respectively. Infection rates of the samples were determined as described above.

Statistics analysis

All statistical analyses were performed using GraphPad Prism software v5.0. Kaplan–Meier survival curves were compared using log-rank test. Statistical significance between treatments was analyzed using Student's 2-tailed *t*-test and indicated as follows: ns, not significant ($P \geq 0.05$); *, $0.01 \leq P < 0.05$; **, $P < 0.01$; ***, $P < 0.001$.

References

1 Zhao FF, Xu YF, Lavillette D, Zhong J, Zou G, Long G. Negligible contribution of M2634V substitution to ZIKV pathogenesis in AG6 mice revealed by a bacterial promoter activity reduced infectious clone. *Sci Rep-Uk* 2018; 8.

2 Antoniou AN, Watts C. Antibody modulation of antigen presentation: positive and negative effects on presentation of the tetanus toxin antigen via the murine B cell isoform of Fc gamma RII. *Eur J Immunol* 2002; 32:530-540.

3 Chan KR, Zhang SLX, Tan HC *et al.* Ligation of Fc gamma receptor IIB inhibits antibody-dependent enhancement of dengue virus infection. *P Natl Acad Sci USA* 2011; 108:12479-12484.

4 Block OKT, Rodrigo WWSI, Quinn M, Jin X, Rose RC, Schlesinger JJ. A tetravalent recombinant dengue domain III protein vaccine stimulates neutralizing and enhancing antibodies in mice. *Vaccine* 2010; 28:8085-8094.

5 Rong Z, Zhigang Y, Yuyan W *et al.* Isolation of a 2019 novel coronavirus strain from a coronavirus disease 19 patient in Shanghai. *JOURNAL OF MICROBES AND INFECTIONS* 2020; 15:111-121.

6 Ye X, Ku Z, Liu Q *et al.* Chimeric virus-like particle vaccines displaying conserved enterovirus 71 epitopes elicit protective neutralizing antibodies in mice through divergent mechanisms. *J Virol* 2014; 88:72-81.

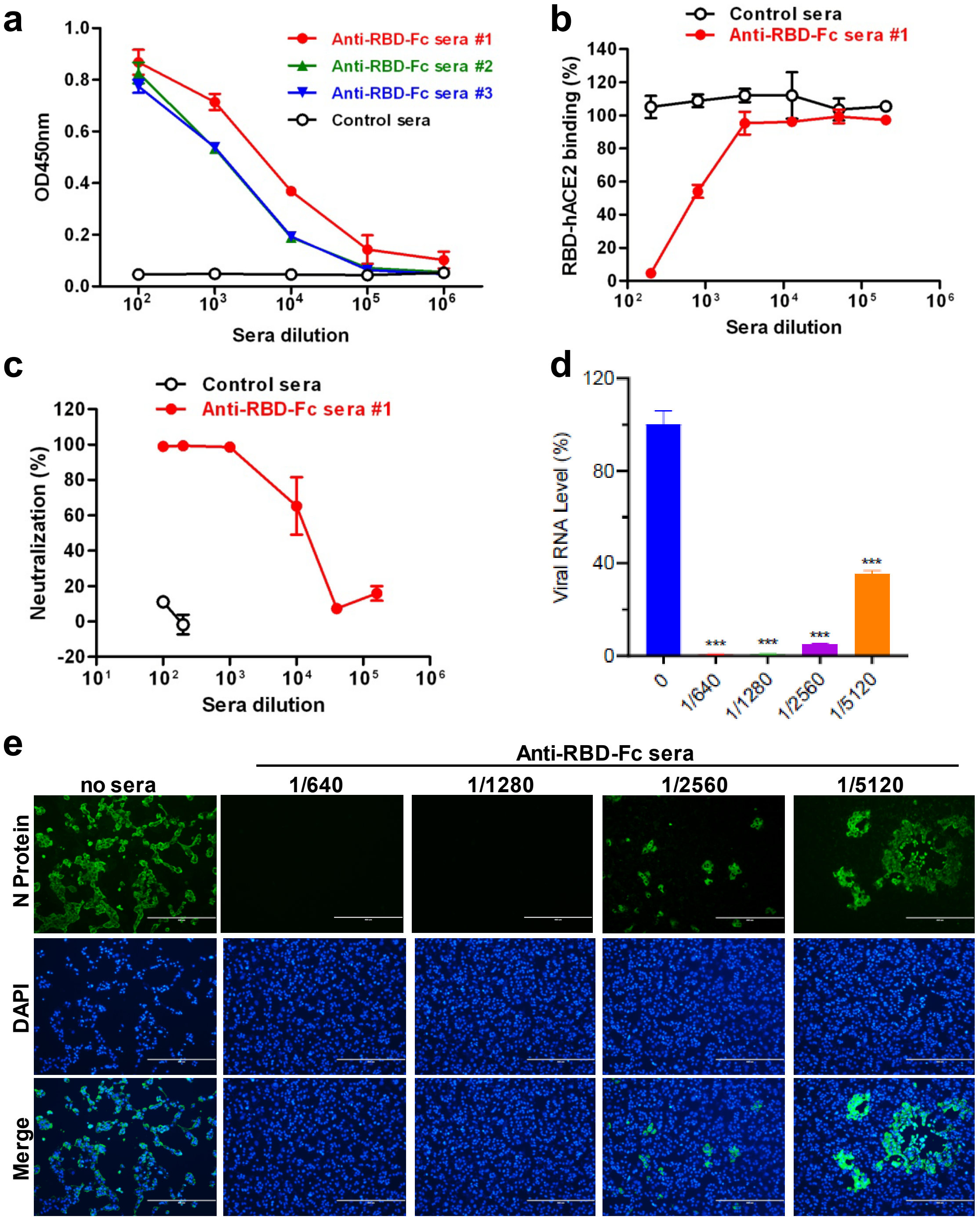

Fig. S1. Immunization with recombinant RBD-Fc fusion protein potently elicited SARS-CoV-2 neutralizing antibodies in mice. a RBD-binding activities of the sera from the three RBD-Fc-immunized mice and the control (naïve) mouse. The sera were serially diluted and then analyzed by ELISA with recombinant SARS2-RBD protein as the coating antigen. Data shown are means and SD of triplicate wells. **b** Inhibitory effect of the anti-RBD-Fc sera on the RBD/ACE2 interaction. The anti-RBD-Fc sera #1 and the control sera were serially diluted and then subjected to ACE2 competition ELISA. Data shown are means and SD of triplicate wells. **c** Neutralization potency of the antisera against SARS-CoV-2 pseudovirus infection.

The antisera were serially diluted and then evaluated for neutralization of SARS-CoV-2 spike-pseudotyped retrovirus. Results from three independent experiments are shown. **d** Neutralization potency of the antisera against authentic SARS-CoV-2 infection. Serially diluted antisera were subjected to neutralization assay with SARS-CoV-2 strain nCoV-SH01 as the challenge virus. Data shown are means and SD of triplicate wells. Significant differences were calculated using student's two-tail t test and shown as: ***, $P < 0.001$. **e** Neutralization of authentic SARS-CoV-2 infection revealed by immunofluorescent staining. Live SARS-CoV-2 virus was incubated with or without serially diluted anti-RBD-Fc sera for 1 hr at 37°C and then added to preseeded VeroE6 cells. After two days, the cells were fixed and then stained sequentially with the N protein-specific primary antibody and a corresponding secondary antibody. Prior to examination under a fluorescent microscope, the cells were briefly stained with DAPI. Representative images are shown. Bar = 400 μm.

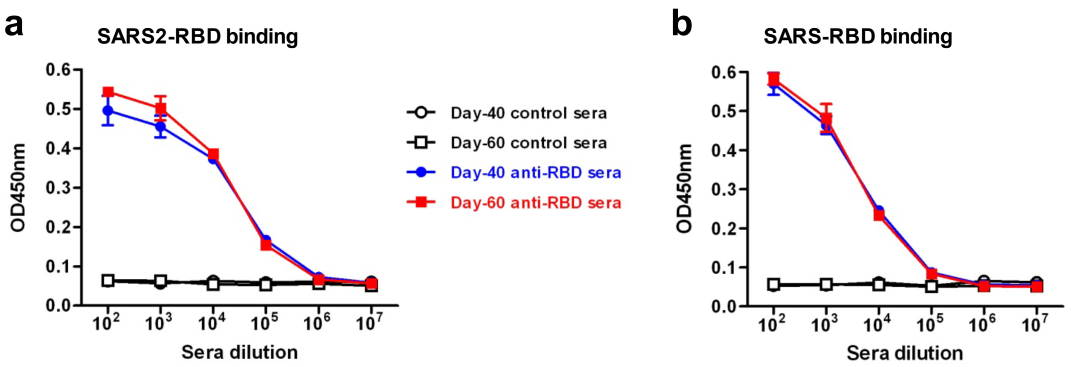

Fig. S2. Comparison of binding activities of the day-40 and day-60 anti-RBD sera pools. The indicated antisera were serially diluted and analyzed by ELISA with (**a**) SARS2-RBD or (**b**) SARS-RBD proteins as the coating antigen. Data shown are mean OD450nm values and SD of triplicate wells.

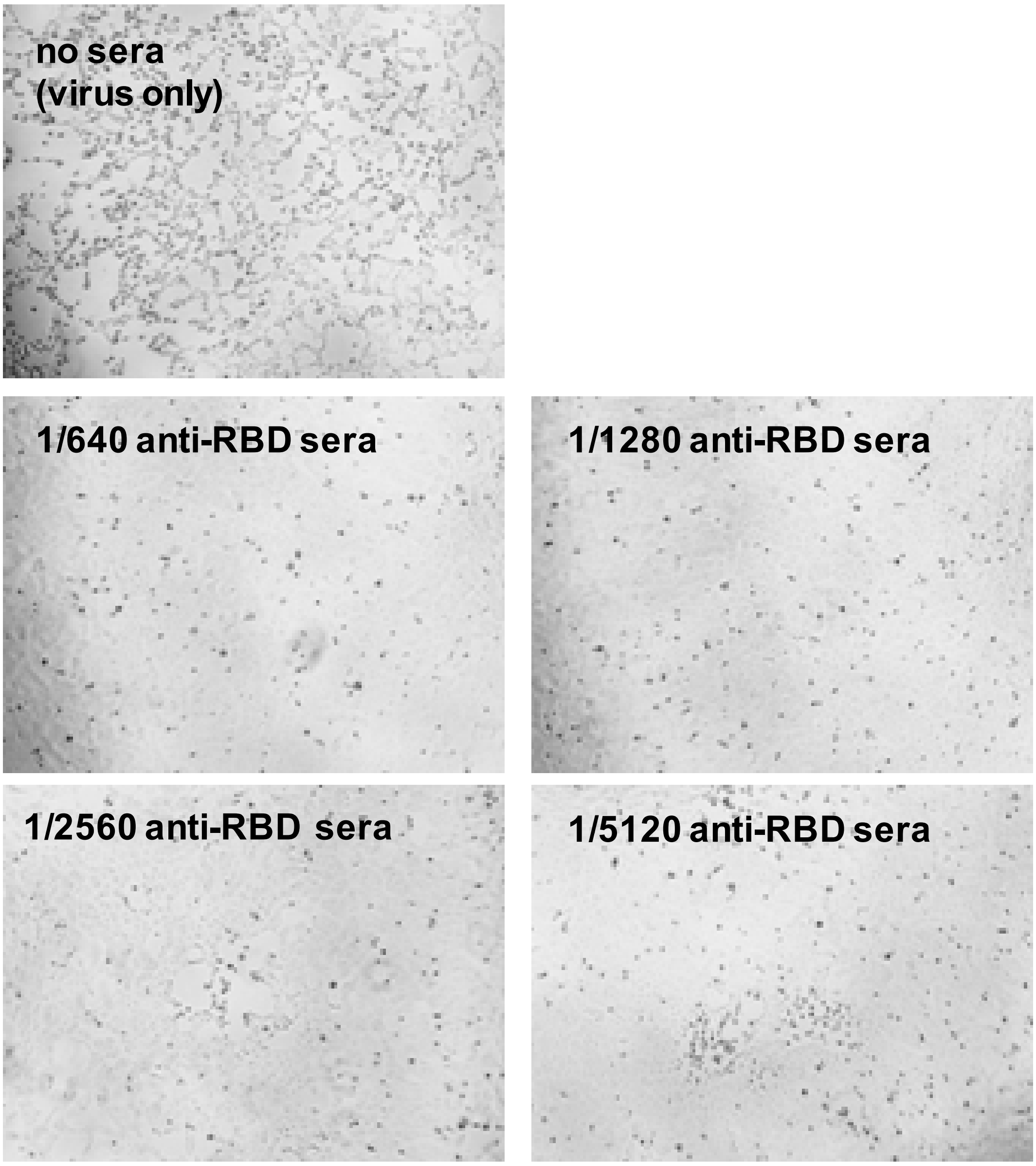

Fig. S3. Treatment with the anti-RBD sera inhibited SARS-CoV-2 infection-triggered CPE. VeroE6 cells were inoculated with mixtures of the authentic SARS-CoV-2 virus and serially diluted anti-RBD sera. The cells were checked daily for CPE. Data presented are images taken at 48 hours post-infection. The test concentrations of the anti-RBD sera are indicated (0, no anti-RBD sera).

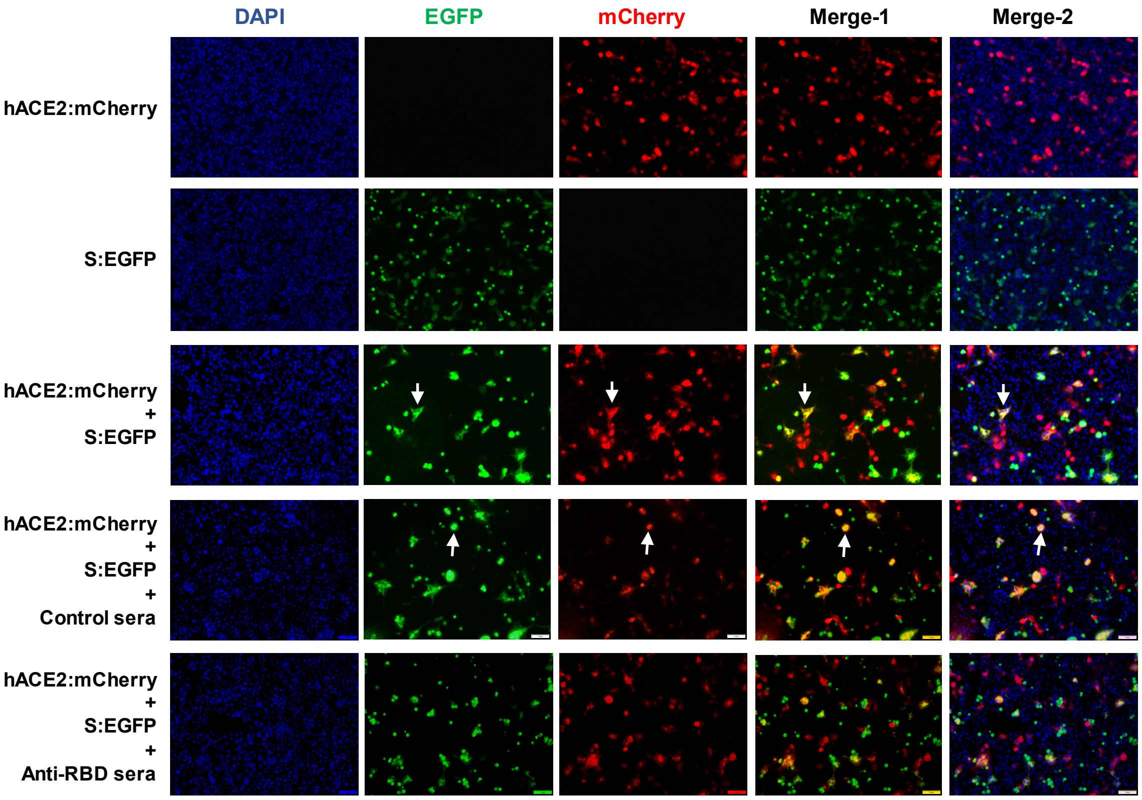

Fig. S4. Cell-cell fusion assay. HEK 293T cells were separately transfected with a plasmid encoding the SARS2-S:EGFP fusion protein (pcDNA-S:EGFP) or with a plasmid encoding the hACE2:mCherry fusion protein (pcDNA-hACE2:mCherry). One day later, equal amount of the pcDNA-S:EGFP-transfected and pcDNA-hACE2:mCherry-transfected cells were mixed and then cultured for 24 hrs. Unmixed cells were set aside as controls. To determine the antisera's blockade effects pcDNA-S:EGFP-transfected cells were treated with serially diluted antisera for 1 hr at 37℃ before mixing with pcDNA-hACE2:mCherry-transfected cells. After co-culture for 24 hrs, the cells were fixed, stained with DAPI and examined under a fluorescent microscope. Representative images are shown. Blue signal, DAPI; green signal, S:EGFP; red signal, hACE2:mCherry; Merge 1, merge of the green and red channels; Merge 2, merge of the blue, green and red channels. Bar = 1 mm.

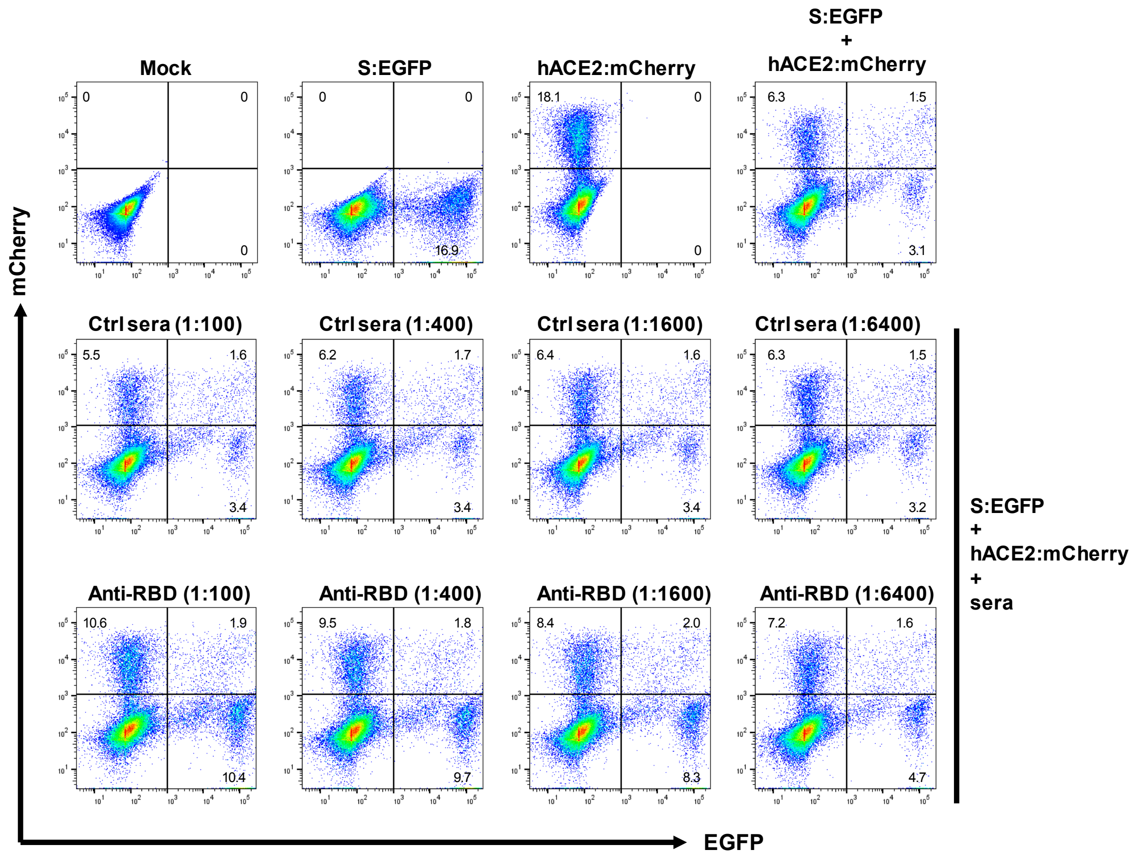

Fig. S5. Inhibition of SARS2-S-mediated cell-cell fusion by anti-RBD sera. HEK 293T cells transiently expressing SARS2-S:EGFP fusion protein were incubated with the indicated dilutions of antisera for 1 hr at 37°C and then mixed with HEK 293T cells transiently expressing hACE2:mCherry, followed by co-culture for 24 hours. Cells without antisera treatment were set as the control. The cell samples were analyzed by flow cytometry. Representative flow cytometry graphs are shown. For a given sample, its cell-cell fusion efficiency was determined as the ratio of the dual-fluorescence cells to the EGFP-only cells.

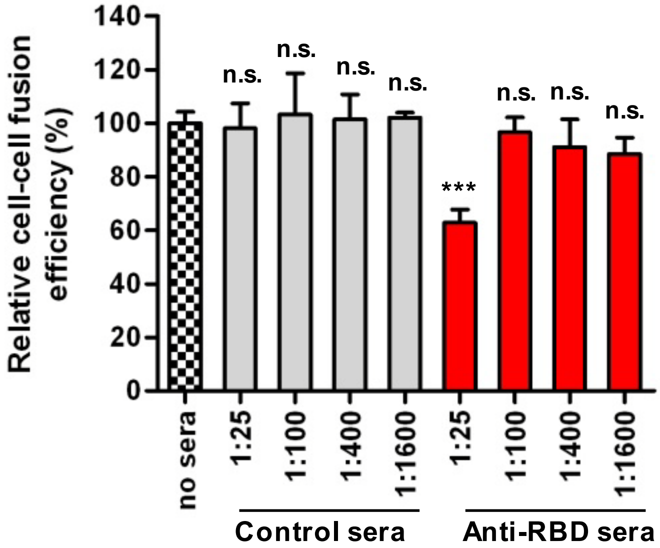

Fig. S6. Inhibition of SARS-S-mediated cell-cell fusion by the anti-RBD sera. HEK 293T cells transiently expressing SARS-S:EGFP fusion protein were incubated with the indicated dilutions of antisera for 1 hr at 37°C and then mixed with HEK 293T cells transiently expressing hACE2:mCherry, followed by co-culture for 24 hours. Cells without antisera treatment were set as the control. The cell samples were analyzed by flow cytometry. Representative flow cytometry graphs are shown. For a given sample, its cell-cell fusion efficiency was determined as the ratio of the dual-fluorescence cells to the EGFP-only cells.
